# Toward a Greater Contextualization of Music and the Arts in Aging and Cognitive Disorders

**DOI:** 10.1093/geroni/igaa057.3084

**Published:** 2020-12-16

**Authors:** Sophie Lee, Hilary Moss, Desmond O’Neill

**Affiliations:** 1 University of Limerick, Limerick, Limerick, Ireland; 2 Irish World Academy of Music and Dance, Limerick, Limerick, Ireland; 3 Trinity College Dublin, Dublin, Dublin, Ireland

## Abstract

Research suggests that group music-making can improve well-being and cognitive function in people with dementia and their family carers. The importance of the music facilitator’s role is recognised. However, empirical studies rarely capture their experiences and perspectives. Semi-structured interviews were conducted with three music therapists and three community musicians with specialisms in dementia care. The interviews sought to gain a detailed understanding of their work with people with dementia. Interpretative Phenomenological Analysis revealed eight super-ordinate themes: (1) benefits of music-making for people with dementia; (2) challenges of working with people with dementia; (3) involving family carers; (4) musical content; (5) impact of the facilitator; (6) developing field of Arts and Health; (7) work as a privilege; and (8) potential for misuse of music. This study provides a useful basis from which to further develop concepts for the amelioration of people living with dementia and their families.

